# Functional Analysis of a Frontal miRNA Cluster Located in the Large Latency Transcript of Pseudorabies Virus

**DOI:** 10.3390/v14061147

**Published:** 2022-05-26

**Authors:** Weronika Hoffmann, Andrea D. Lipińska, Krystyna Bieńkowska-Szewczyk

**Affiliations:** Laboratory of Virus Molecular Biology, Intercollegiate Faculty of Biotechnology, University of Gdańsk, Abrahama 58, 80-307 Gdańsk, Poland; weronika.hoffmann@biotech.ug.edu.pl

**Keywords:** pseudorabies virus, Large Latency Transcript, miRNA cluster, IE180, EP0, glycoprotein E

## Abstract

MicroRNAs (miRNAs) have been identified as a class of crucial regulators of virus-host crosstalk, modulating such processes as viral replication, antiviral immune response, viral latency, and pathogenesis. Pseudorabies virus (PRV), a model for the study of alphaherpesvirus biology, codes for 11 distinct miRNAs mapped to the ~4.6 kb intron of Large Latency Transcript (LLT). Recent studies have revealed the role of clusters consisting of nine and eleven miRNA genes in the replication and virulence of PRV. The function of separate miRNA species in regulating PRV biology has not been thoroughly investigated. To analyze the regulatory potential of three PRV miRNAs located in the frontal cluster of the LLT intron, we generated a research model based on the constitutive expression of viral miRNAs in swine testis cells (ST_LLT [1–3] cell line). Using a cell culture system providing a stable production of individual miRNAs at high levels, we demonstrated that the LLT [1–3] miRNA cluster significantly downregulated IE180, EP0, and gE at the early stages of PRV infection. It was further determined that LLT [1–3] miRNAs could regulate the infection process, leading to a slight distortion in transmission and proliferation ability. Collectively, our findings indicate the potential of LLT [1–3] miRNAs to retard the host responses by reducing viral antigenic load and suppressing the expansion of progeny viruses at the early stages of infection.

## 1. Introduction

Pseudorabies virus (PRV, also known as suid herpesvirus type 1, SuHV-1) belongs to the *Alphaherpesvirinae* subfamily and is an etiologic agent of Aujeszky’s disease. PRV causes neurological, respiratory, and reproductive disease in pigs, its natural hosts [1]. Despite successful eradication campaigns using inactivated or attenuated live PRV vaccines, pseudorabies outbreaks still occur in swine populations, causing huge economic losses in the pig industry worldwide [2,3]. Although humans are thought to be resistant to PRV infection, recent studies indicate that PRV may be a zoonotic pathogen causing encephalitis [4,5]. PRV is a commonly used model for the studies on the pathogenesis and molecular biology of herpesviruses. It is a tool for neural circuit tracing [6].

The alphaherpesviral life cycle is primarily controlled at the level of transcription. PRV genes are subdivided into three types: immediate-early, early, and late [7]. PRV encodes the sole Immediate Early 180 (IE180) gene, which is transcribed independently of de novo viral protein synthesis. The IE180 protein is the key viral activator of the productive (lytic) infection, launching the transcription cascade of another viral gene expression [8]. The gene expression of PRV Early Protein 0 (EP0) is dependent on IE180. EP0 acts in cooperation with IE180 to activate early and late gene transcription, which are necessary for DNA replication and the production of structural proteins required for viral morphogenesis [7,9]. In pigs surviving the lytic infection, the virus can develop a lifelong latency stage in the trigeminal ganglia (TG) of the peripheral nervous system, which is a feature common to all herpesviruses [6,10]. During latency, there is no virus progeny production, and the transcription of the viral genome is limited to a segment of a non-coding region called the Latency Associated Transcript (LAT) [11]. The LAT locus has been reported to function as a primary precursor of clustered microRNAs (miRNAs, miRs) for several alphaherpesviruses [12,13].

MiRNAs are small, non-coding RNAs of 21-24-nt that act as post-transcriptional regulators of mRNA expression, either via the induction of transcript degradation or by the suppression of translation [14,15]. Gene expression is regulated mainly by proteins, but the regulation by miRNAs seems to have some advantages: they require relatively little coding capacity, they are not antigenic, and the evolution of miRNAs can occur more easily [16]. The Epstein-Barr virus, a gammaherpesvirus, is the first virus reported to encode miRNAs [17]. Recent discoveries indicate that these small, regulatory RNAs can contribute to the repertoire of host-pathogen interactions during viral infection. Viral miRNAs have been reported to control the environment of the infected cell by regulating viral and host gene expression, which leads to the modulation of viral replication, latency control, or immune evasion [18,19,20].

One of the most abundant transcripts during latent PRV infection is the Large Latency Transcript (LLT), an 8.4 kb polyadenylated RNA with a 4.6 kb stable intron [21]. The LLT is involved in maintaining the latent stage of PRV infection [6] but is also expressed during productive infection of cultured cells [22]. Using deep sequencing techniques, a cluster of eleven viral miRNAs has been identified in the porcine kidney (PK15) cells during productive infection [23]. A similar cluster of five viral miRNAs could be detected in PRV-infected immature dendritic cells [24], indicating that the intron of the LLT functions as the primary precursor of PRV miRNAs. In silico predictions suggest that PRV LLT-related miRNAs play an important role in controlling both viral self-regulatory network and host-virus interactions [23,25,26,27]. To date, the functional analyses of particular PRV miRNA genes have been performed only for prv-miR-LLT7-5p and prv-miR-LLT11a [28,29]. Therefore, the functional validation of the separate LLT intron fragments requires further investigation to obtain a more detailed insight into the miRNA regulatory network.

In the present study, we established a research model based on the stable overexpression of miRNAs of the European PRV-NIA-3 strain in epithelial cells for the observations of phenotypic changes after PRV lytic infection. A cell culture system providing a stable production of individual miRNAs at high levels can be a useful tool for highlighting miRNA-specific functions and differentiating viral miRNA activity from other infection-related mechanisms. The constructed cell line codes for the first three miRNA genes mapped to the LLT intron. Our study demonstrated that the IE180 gene, which was often indicated as a target for viral miRNAs, was downregulated by the overexpression of the frontal miRNA cluster from the LLT intron. We also verified the influence of these miRNAs on viral replication kinetics and propagation. This investigation provides presumptive evidence on the role of analyzed miRNAs in the cell-to-cell spread process. We hypothesize that these miRNAs may contribute to the control of the course of PRV infection, presumably via the adjustment of viral progeny assembly.

## 2. Materials and Methods

### 2.1. Cell Lines and Virus Infection

Swine testis cells (ST, kindly provided by Dr. H. Favoreel, Department of Virology, Parasitology, and Immunology, Faculty of Veterinary Medicine, Ghent University, Belgium) were cultured in Minimum Essential Medium Eagle (MEME, Corning, Corning, NY, USA) supplemented with 10% fetal bovine serum (FBS, Thermo Scientific, Waltham, MA, USA). GP2-293 retrovirus packaging cells (Takara/Clontech, Kusatsu, Japan) were cultivated in Iscove’s Modified Dulbecco Medium (IMDM, Merck/Sigma, Darmstadt, Germany) supplemented with 10% FBS and 2 mM L-glutamine (Thermo Scientific, Waltham, MA, USA). Cells were grown in a 5% CO_2_ environment at 37 °C with the addition of an Antibiotic-Antimycotic solution (Thermo Scientific, Waltham, MA, USA).

Wild type PRV reference strain Northern Ireland Aujeszky-3 (NIA-3, kindly provided by Dr. Liesbeth Jacobs, Institute for Animal Health and Science, Lelystad, The Netherlands) was propagated and titrated by a plaque assay on ST cells. ST cell lines were infected with PRV at a multiplicity of infection (MOI) of 1 and harvested at different time points as follows: for immunoblotting and FACS analysis at 3, 5, and 7 h post-infection (hpi); for immunofluorescence at 1, 5, and 7 hpi; for miRNA and transcript analysis at 1, 3, 5, 7, 12, and 24 hpi. After two hours of incubation, the virus inoculum was replaced with a fresh culture medium.

### 2.2. Generation of an ST Cell Line for Constitutive PRV miRNA Expression

A cluster of PRV miRNAs encompassing a region from prv-miR-LLT1 to prv-miR-LLT3 was introduced to ST cells using retroviral gene transfer. The sequence coding for the cluster of PRV miRNAs precursors (pre-miRNAs) was PCR-amplified from isolated viral DNA and inserted into the BamHI/EcoRI sites of the pLZRS-IRES-GFP vector [30] upstream of the internal ribosome entry site (IRES) and downstream of the 5′LTR Moloney leukemia retrovirus RNA polymerase II-dependent promoter [31]. PCR reactions were performed using the KAPA HiFi DNA polymerase (Kapa Biosystems, Wilmington, MA, USA) with 5% DMSO enhancing GC-reach products amplification. PCR primers used for prv-miR-LLT [1–3] sequence production are listed in Table 1. The pLZRS-IRES-GFP-miR-LLT [1–3] construct, in combination with the pVSV-G envelope vector (Cell Biolabs, San Diego, CA, USA), was used for calcium phosphate-based transfection (CalPhos kit, Clontech/Takara, Kusatsu, Japan) of the GP2-293 packaging cells for retrovirus production. The retrovirus-containing medium was collected after 48 h and used for ST cell transduction in the presence of 0.01 mg mL^−1^ polybrene (Merck). GFP-positive cells were sorted using a FACSCalibur flow cytometer (Becton Dickinson, Franklin Lakes, NJ, USA) to generate ST_LLT [1–3] cell line > 98%-positive for GFP.

### 2.3. Cell Viability Assay

To compare the cell viability between ST_LLT [1–3] and control ST cells, they were seeded in 8-fold repeats in a 96-wells plate. Cells of both lines, treated with 10% DMSO, were used as a control. After 24 h incubation, 20 μL of combined MTS and PMS solutions from the CellTiter 96^®^ AQueous One Solution Cell Proliferation Assay (Promega, Madison, WI, USA) were added to each well. After 2 h incubation, the colorimetric signal was measured using the Infinite M200 plate reader (Tecan, Männedorf, Switzerland).

### 2.4. RNA Extraction and Quantitative Reverse Transcription PCR (RT-qPCR)

Total RNA fractions were prepared with the use of the TRI Reagent (Molecular Research Center, Cincinnati, OH, USA) for miRNA and mRNA quantification by RT-qPCR. To analyze the expression of prv-miR-LLT1, prv-miR-LLT2, and prv-miR-LLT3 in the ST_LLT [1–3] stable cell line and in ST cells infected with PRV, 0.5 μg of total RNA was subjected to polyadenylation and reverse transcription using the Mir-X miRNA First Strand Synthesis Kit (Clontech/Takara, Kusatsu, Japan). Next, synthesized cDNAs were diluted to a concentration of 25 ng μL^−^^1^, and 50 ng was applied as a template to RT-qPCR using the SYBR Premix Ex Taq II (Tli RNase H Plus, Clontech/Takara, Kusatsu, Japan). Mature miRNA quantification was performed using miRNA-specific forward primers and the universal reverse primer mRQ, complementary to poly(T) adapter (supplied with the Mir-X miRNA First Strand Synthesis Kit). Sequences of miRNA-specific oligonucleotides were designed based on miRbase.org (accession numbers in brackets): prv-miR-LLT1-(MIMAT0025304), prv-miR-LLT-(MIMAT0025305) and prv-miR-LLT3-(MIMAT0025306). To ensure the binding of those oligos to mature miRNAs and to avoid their hybridization to miRNA precursors, a double A residue was added to the 3′-end of each primer to provide the binding to poly(T) region of mature miRNA cDNAs. The relative gene expression of PRV miRs was calculated by the ∆∆C_t_ method. To normalize the amount of total RNA in each reaction, U6 small nuclear RNA was used as an internal control. The reactions were run in a LightCycler 480 instrument (Roche) using the following conditions: 95 °C 30 s and (95 °C 5 s, 62 °C 20 s) for 45 cycles. After the amplification, reaction products were subjected to the dissociation program (95 °C 0 s, 55 °C 15 s, 95 °C 0 s ramp rate 0.1 °C per s) for melting curve analysis.

To detect PRV transcripts, 1 µg of total RNA was reverse transcribed using SuperScript IV Reverse Transcriptase (Invitrogen) and the oligo d(T)_20_ primer. RT-qPCR was performed using SG/ROX qPCR Master Mix (Eurx, Gdańsk, Poland) and specific primers for genes encoding IE180, EP0, and gE. 28S ribosomal RNA was used as an internal control. The relative level of PRV mRNA expression was calculated by the ∆∆C_t_ method.

### 2.5. Viral Replication Kinetics

For the estimation of replication kinetics, one-step growth curve analysis and plaque size calculation were performed. For plaque size analysis, cell monolayers were inoculated with 100 plaque-forming units (pfu) in a 6-well plate. After 48 h incubation, cells were fixed with 4% paraformaldehyde in a phosphate-buffered saline (PBS, Thermo Scientific, Waltham, MA, USA) and immunostained for 1 h with mouse anti-PRV glycoprotein E monoclonal antibody (a kind gift from Dr. Hanns-Joachim Rziha, Friedrich Loeffler Institut, Federal Research Institute for Animal Health, Tübingen, Germany) diluted at 1:100. After 1 h incubation with horseradish peroxidase (HRP)-conjugated secondary antibodies, plaques were visualized using the NovaRED Substrate (Vector Laboratories, Burlingame, CA, USA). Plaques were imaged under a microscope at a 200× magnification and plaque areas were calculated using the LUCIAImage software (Laboratory Imaging, Praha, Czech Republic).

One-step growth experiment was performed as follows: cells were infected with the virus at an MOI of 1; after two-hour incubation, cells were washed with PBS, incubated with citrate buffer (40 mM sodium citrate, 10 mM KCl, 135 mM NaCl, pH 3.0) for 2 min to inactivate non-internalized viruses [33], and finally washed three times with PBS to remove PRV virions from the cell surface. Next, a fresh medium was applied, and cells were harvested at 3, 5, 7, 12, and 24 hpi for intracellular virus titration. For extracellular virus titration, the viral inoculum was collected at the same time points. Viral titers were defined on ST cells by a plaque assay.

### 2.6. Protein Extraction and Immunoblotting

Cells were lysed in the Cell Lytic M buffer (Merck/Sigma, Darmstadt, Germany) supplemented with the cOmplete mini protease inhibitor cocktail (Roche). Total protein concentration was measured at 280 nm using the DS-11 Spectrophotometer (DeNovix, Wilmington, DE, USA). Cell lysates were resolved by 8% or 10% SDS-PAGE and transferred to PVDF membrane (Merck Millipore, Burlington, MA, USA), which was subsequently blocked with 5% bovine serum albumin (BSA, Merck/Sigma, Darmstadt, Germany). Primary antibodies were incubated for one hour at room temperature (RT). Goat B1B2 anti-PRV IE180 polyclonal antibody was used at a dilution of 1:5000 (from Dr. Hanns-Joachim Rziha). Mouse anti-PRV gE mAb was diluted at 1:2000. Mouse anti-β-actin monoclonal antibody (Novus Biologicals) was diluted at 1:5000. This was followed by HRP-conjugated secondary antibodies for 1 h at RT. The signal was detected by chemiluminescence using the Clarity Max Western ECL Substrate (Bio-Rad, Hercules, CA, USA) and the Alliance Q9 Mini instrument (Uvitec, Cambridge, UK). Densitometric analysis was performed using the Uvi Band software (Uvitec).

### 2.7. Immunofluorescence Imaging

Cells were grown on micro cover glass for 24 h and then infected with PRV. After indicated time points, cells were fixed with 4% paraformaldehyde, permeabilized with 0.2% Triton X-100 in PBS for 7 min, and stained for 1 h with mouse anti-PRV gE mAb diluted at 1:100. Next, cells were incubated for 1 h with Alexa 546-conjugated goat anti-mouse antibody (Thermo-Molecular Probes, Waltham, MA, USA) used at a dilution of 1:3000. After staining, cells were analyzed using the Leica TCS SP8X confocal laser scanning microscope (Leica, Wetzlar, Germany).

### 2.8. Flow Cytometry

Cell surface expression of PRV gE after PRV infection was determined by indirect immunofluorescence using primary anti-PRV gE mAb diluted at 1:100, staining for 1 h, and Alexa 633-conjugated goat anti-mouse antibody (Thermo-Molecular Probes) diluted at 1:1000, staining for 1 h. Cells were kept on ice during the whole procedure and subsequently analyzed using the Guava easyCyte flow cytometer (Merck) and the InCyte software.

### 2.9. Statistical Data Analysis

The data were statistically analyzed using the unpaired *t*-test of Microsoft Excel.

## 3. Results

### 3.1. Generation and Characterization of the ST Cell Line Constitutively Expressing PRV LLT [1–3] miRNAs

**Figure 1:**
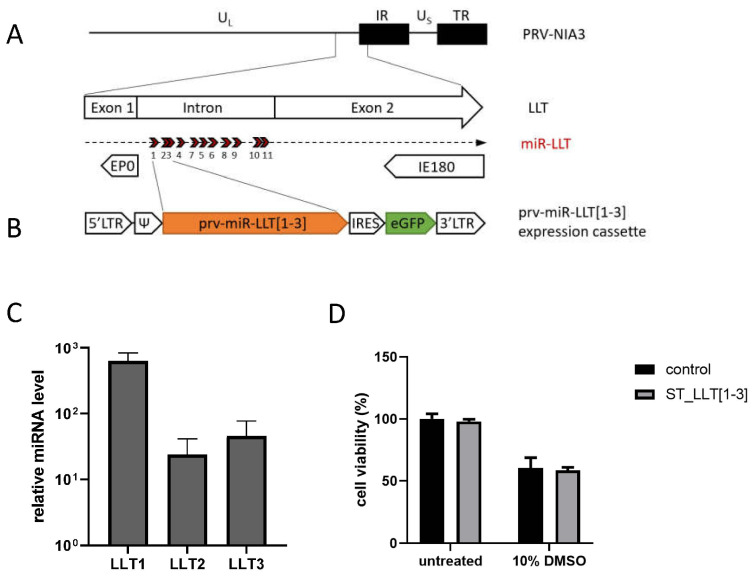
Characteristics of the ST_LLT [1–3] cell line constitutively expressing PRV miRNAs. (**A**) Schematic map of PRV-NIA-3 genome containing unique (UL and US) and inverted repeat (IR and TR) sequences. The enlarged section shows the border region between UL and IR, with loci for the EP0 and IE180 genes. The loci for prv-miR-LLTs (numbered from 1 to 11) are indicated by arrows. (**B**) Schematic map of the prv-miR-LLT [1–3] expression cassette. The LLT [1–3] miRNA cluster was introduced to the pLZRS-IRES-GFP retroviral vector, containing terminal repeats (5′LTR and 3′LTR), a packaging signal (Ψ), an internal ribosome entry site (IRES), followed by eGFP-encoding sequence. (**C**) Overexpression of PRV miRNAs in ST_LLT [1–3] cell line was confirmed by RT-qPCR. Fold differences of prv-miR-LLT1-3p, prv-miR-LLT2-5p, and prv-miR-LLT3-3p levels, relative to the U6 internal control, were determined with RT-qPCR using the ∆∆C_t_ method (with normalization of the relative miRNA expression to that of control ST cells). miRNA expression is depicted as mean values from three independent quantifications. (**D**) The PRV miRNAs effect on cell viability and proliferation was measured by the cell proliferation assay. The ST_LLT [1–3] or control ST cells were analyzed in 8-fold repeats (means with standard deviations are provided). Both cell lines were also treated with 10% dimethyl sulfoxide (DMSO) as a control.

To investigate the regulatory potential of PRV-encoded miRNAs on virus gene expression and replication, we have established a research model based on the swine testicle (ST) cell line with constitutive overexpression of PRV miRNAs in the absence of infection. We decided to study the function of three first PRV miRNAs, which originate from the same primary precursor (pri-miRNA), and, as a part of a single cluster, are expressed simultaneously. ST are spontaneously immortalized cells with fibroblast-like morphology characterized as an immature porcine Sertoli cell line [34]. These cells are frequently used for PRV propagation and experiments [35,36]. ST cells, with the proposed mesenchymal-like status, may exhibit unique features, varying from the porcine epithelial PK15, porcine dendritic, or mouse neuroblast neuro-2A cells where previously PRV miRNA expression and function have been established [23,24,25,26,27]. We used the retrovirus transduction system to obtain a stable ST cell line that constitutively expresses PRV miRNA cluster LLT [1–3]. We chose this model of study to minimize the risk of toxicity and variability of results resulting from plasmid or RNA transfection that could influence subsequent virus infection studies. The pLZRS-IRES-GFP retroviral vector (Figure 1B) contains a heterologous retrovirus promoter (5′LTR) and an eGFP-encoding sequence, enabling cell sorting. The recombinant retrovirus codes for the sequence of nucleotides 97,486 to 98,687 of the PRV-NIA-3 genome (GenBank accession no. KU900059) [37], which includes three out of 11 miRNA genes (described in [23]): prv-miR-LLT1-3p (LLT1), prv-miR-LLT2-5p (LLT2), and prv-miR-LLT3-3p (LLT3), together with the flanking sequences. The incorporated region is located at the right arm of the unique long segment (U_L_) within the intron of the Large Latency Transcript (LLT) of the PRV genome (Figure 1A), which is antiparallel to and overlaps the genes of two important viral activators of transcription: Immediate Early Protein 180 (IE180) and Early Protein 0 (EP0) [21]. We decided to generate a cell line clustering LLT1, LLT2, and LLT3, as LLT2 and LLT3 are produced from the jointed hairpin precursors [23].

The RT-qPCR verified the production of three PRV miRNAs in the constructed ST_LLT [1–3] cell line. The expression of miRNAs relative to the U6 endogenous control was determined using the ∆∆C_t_ method. Average fold differences were estimated by normalizing the relative expression (∆C_t_ values) to those of the control ST cells (background control). The LLT1, LLT2, and LLT3 signals were found to be approximately 600-, 20-, and 50-fold over the background, respectively (Figure 1C). The RT-qPCR results confirmed the overexpression of viral miRNAs in the constructed cell line, with the predominant production of LLT1.

To test whether PRV miRNAs stably produced in the ST_LLT [1–3] cell line affect the cell viability, the cell proliferation assay was performed. We observed no significant difference in cell viability between both cell lines, which indicates that PRV miRNAs were not toxic and did not affect the host gene expression required for cell proliferation or maintenance (Figure 1D).

### 3.2. Expression Profiles of LLT [1–3] miRNAs during PRV Infection

**Figure 2:**
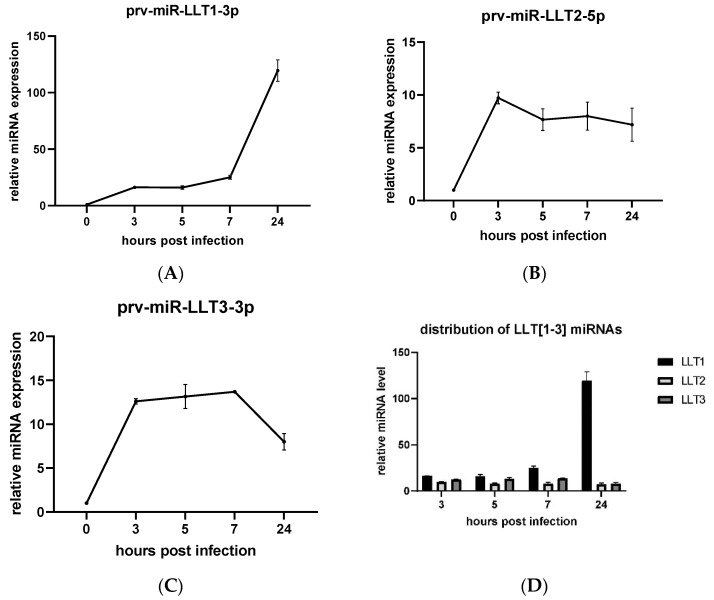
Production of PRV LLT [1–3] miRNAs during PRV infection in cultured cells. The ST cells were infected with PRV at the multiplicity of infection (MOI) of 1 and collected at 3, 5, 7, and 24 hpi. The expression profiles of (**A**) LLT1, (**B**) LLT2, and (**C**) LLT3 in ST cells were assessed by RT-qPCR against mock-infected ST cells (time point 0). (**D**) Pattern of PRV LLT [1–3] miRNAs appearance in the course of infection. The data were reported as mean ± SD; the experiments were performed in triplicates.

We also asked a question about the expression profile of those three PRV miRNAs during the productive infection. The levels of viral miRNAs in the PRV-infected ST cells were determined using RT-qPCR. We could detect all three miRNAs as early as 3 hpi. The quantities of LLT1 increased as the infection progressed, reaching the maximum at 24 hpi. The abundance of LLT1 in the infected ST cells increased 16-fold within the first 5 hpi, and 25- and 120-fold at 7 and 24 hpi, respectively, compared to mock-infected control ST cells (Figure 2A). The level of LLT2 increased 8-fold towards the control and was quite stable during the course of infection (Figure 2B). The quantities of LLT3 were upregulated during the first seven hours of infection (a 13-fold increase), and afterward significantly downregulated—an 8-fold increase compared to the mock-infected control (Figure 2C). As shown in Figure 2D, the pattern of the LLT [1–3] cluster-derived miRNAs’ appearance during the course of PRV infection was similar, with the predominant expression of LLT1 at 24 hpi.

### 3.3. IE180 and EP0 Expression Is Downregulated by the Overexpression of LLT [1–3] miRNAs

**Figure 3:**
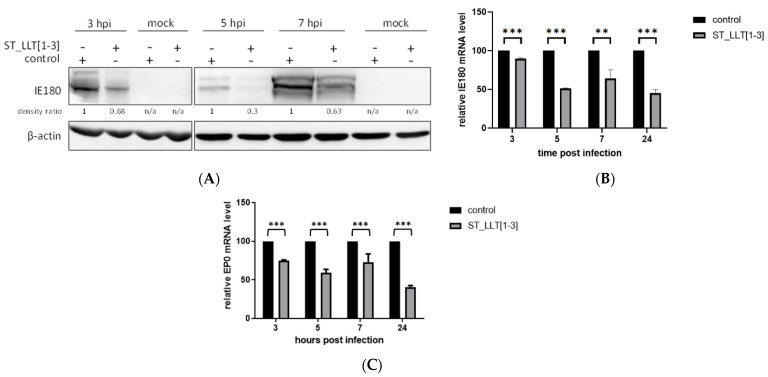
PRV LLT [1–3] miRNAs downregulate the expression of IE180 and EP0. (**A**) The ST_LLT [1–3] cells and control ST cells were infected with PRV and IE180 protein level was determined by immunoblotting: at 3 hpi (160 µg of total protein extract per well), 5 hpi, and 7 hpi (40 µg of total protein extract per well). β-actin was detected as a loading control. (**B**) Densitometric quantifications of IE180 bands (indicated as the density ratio), normalized to the density of corresponding β-actin bands, were calculated between the control and ST_LLT [1–3] cell lines for each time point after PRV infection. n/a, not applicable. The mRNA levels of B. IE180 and (**C**) EP0 were measured by RT-qPCR at the indicated time points in the ST_LLT [1–3] cells and the control ST cells after PRV infection. 28S gene was used as a reference internal control. The experiment was performed in triplicate, data are expressed as the mean ± standard deviation. ** *p* < 0.01, *** *p* < 0.001.

To define the role of miRNAs in viral biology, the identification of target genes is required, which can be a challenging task because miRNAs require only limited complementarity [38]. Based on the bioinformatics analyses, potential target sequences for individual PRV miRNAs were listed by various research groups [23,24,25,26]. The PRV-encoded miRNAs together with the target viral genes, and the host genes, form a complex regulatory network, where many miRNAs, like prv-miR-LLT1-3p and prv-miR-LLT3-3p, target multiple genes. Most of the predictions indicate the 3′UTR of IE180 transcript as a putative target for PRV miRNAs clustered in the LLT intron. Among these miRNAs, prv-miR-LLT1-3p is predominantly predicted to target IE180 mRNA. IE180 is the main regulator of productive infection, activating the transcription of early and late viral genes [8], and its mRNA is synthesized within 40 min of infection [6]. To test whether viral miRNAs produced in the ST_LLT [1,2,3] cell line exhibit the potential to regulate the expression profiles of the IE180 gene, we conducted functional studies at the early stages of the infection. The immunoblotting analysis showed that IE180 could be detected at 3 hpi in ST cells, and the presence of miRNA LLT [1–3] cluster significantly decreased the IE180 level at that time point (Figure 3A). Subsequently, we examined whether the inhibitory effect of viral miRNAs could persist over time. As shown in Figure 3A, at 5 and 7 hpi, the miRNA impact on IE180 production could be still observed. Those observations were confirmed by IE180 transcript detection by RT-qPCR. The PRV-infected ST_LLT [1–3] cells exhibited a decreased level of IE180 mRNA compared to the control ST cells at indicated time points (Figure 3B). These findings indicate that PRV LLT [1–3] miRNAs exhibit regulatory potential over both the transcript and protein levels of IE180 during the PRV life cycle. Next, we asked a question regarding whether LLT [1–3] miRNAs could also affect the expression of EP0, an IE180-dependent early gene whose product acts as a transactivator, in cooperation with IE180, of viral gene transcription [7,9]. Our results demonstrated that PRV miRNAs from the LLT [1–3] cluster also downregulated the levels of EP0 transcript in PRV-infected ST_LLT [1–3] cells compared to the control ST cells (Figure 3C). Due to the lack of specific antibodies, we could not analyze the level of EP0 protein.

### 3.4. Production of gE Is Hampered by LLT [1–3] miRNAs

**Figure 4:**
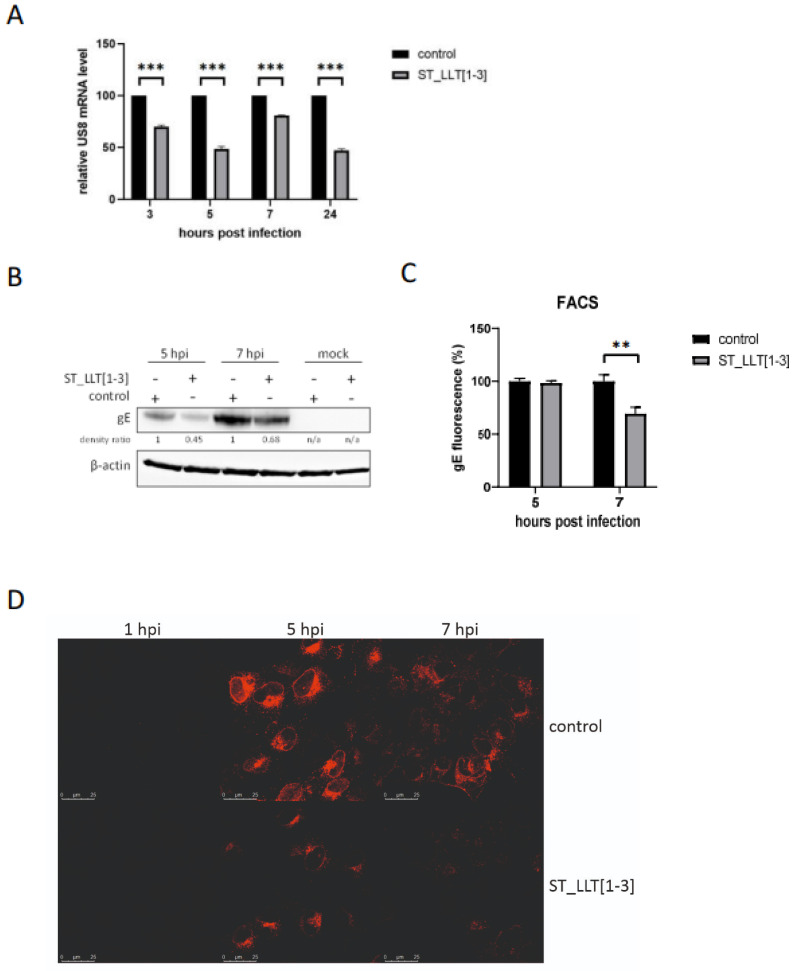
PRV-infected ST_LLT [1–3] cells exhibit decreased gE expression (**A**) The RT-qPCR analysis of US8 transcript in the ST_LLT [1–3] cells infected with PRV, compared to the control ST cells. 28S gene was used as a reference internal control. mRNA expression is depicted as mean values from three independent quantifications. *** *p* < 0.001. (**B**) The immunoblot analysis at 5, and 7 hpi of PRV gE using lysates of the ST_LLT [1–3] cells and the control ST cells infected with PRV. β-actin was detected as a loading control. Densitometric quantifications of gE bands (indicated as the density ratio), normalized to the density of corresponding β-actin bands, were calculated between the control and ST_LLT [1–3] cell lines for each time point after PRV infection; n/a: not applicable. (**C**) The effect of LLT [1–3] miRNAs on the surface expression of gE in ST_LLT [1–3] cells was assessed by flow cytometry at 5, and 7 hpi and compared to uninfected control ST cells. The analysis was performed in triplicates. ** *p* < 0.01. (**D**) The level of subcellular gE in PRV-infected cells was evaluated by immunofluorescence laser scanning confocal microscopy (with the same image collection and analysis settings for all the samples).

As a next step, we investigated whether miRNAs LLT [1–3] demonstrate an impact on the expression of an early-late US8 gene [39], coding for glycoprotein E (gE), an envelope protein contributing to the virulence of PRV [40]. According to the RT-qPCR results presented in Figure 4, the production of gE transcript decreased in the presence of LLT [1–3] miRNAs (Figure 4A). The pattern of gE mRNA inhibition was similar to that of the IE180 transcript, which stands in line with the importance of IE180 for early gene expression. As shown in Figure 4B, the total gE protein level in lysed cells was lower in the ST cells constitutively expressing PRV miRNAs towards the control ST cells at 5 and 7 hpi. Those observations were confirmed by the analysis of gE at the surface of infected cells by flow cytometry. At 7 hpi, gE surface expression was approximately 30% lower in the PRV-infected LLT [1–3] cells compared to the control (Figure 4C). These results were verified by the detection of gE by the immunofluorescence assay. As expected, a significantly lower level of gE was present at the surface of PRV-infected ST_LLT [1–3] cells compared to the infected control ST cells (Figure 4D).

### 3.5. The Effect of LLT [1–3] miR Cluster Overexpression on PRV Replication

**Figure 5:**
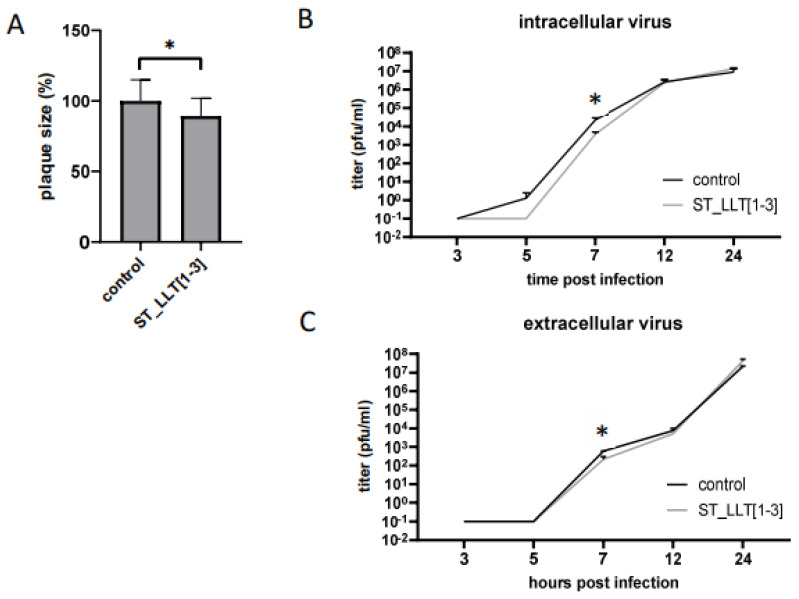
Overexpression of PRV LLT [1–3] miRNAs affects PRV replication kinetics (**A**). The ST_LLT [1–3] cells and the control ST cells were infected with PRV and the plaque sizes of replicating PRV was analyzed. One-step growth kinetics of intracellular (**B**) and extracellular (**C**) virus replicating in the PRV-infected ST_LLT [1–3] cells compared to the control ST cells. The viral titers were determined on the ST cells using the plaque assay; * *p* < 0.05.

Once we established that the overexpression of PRV LLT [1–3] miRNAs influences IE180 and gE levels in the infected ST_LLT [1–3] cells, we asked whether the PRV genes’ downregulation could affect viral replication. As gE functions to mediate cell-to-cell spread in neuronal tissues and cultured epithelial cells [41], we first checked the effect on PRV plaque morphology. The plaque size analysis showed that in the monolayer of the ST_LLT [1–3] cells, PRV generates plaques that are, on average, 10% smaller compared to the control ST cells (Figure 5A). This result implies that the PRV miRNA overexpression slightly limits the viral spread to uninfected neighboring cells.

Because IE180, as a sole transactivator of early genes, is a key player in the viral gene expression and replication, in the next step, we examined how the downregulation of IE180, caused by overexpression of LLT [1–3] miRNAs, could influence the growth kinetics. A one-step growth curve analysis for intracellular virions (Figure 5B) demonstrated that PRV proliferation at 5 hpi in the ST_LLT [1–3] cell line was arrested, while PRV progeny was detectable in ST control cells. At 7 hpi, the viral load was approximately reduced 6-fold in the ST_LLT [1–3] cells. Similarly, the titers of the extracellular progeny PRV virions (Figure 5C) were approximately 3 times lower in the ST_LLT [1–3] cells compared to the control ST cells. The analysis of the growth kinetics suggests a slight delay in the onset of viral production: at 12 hpi, the titers were approximately 15% (for the intracellular virus) and 25% (for the extracellular virus) lower in ST-LLT [1–3] cells compared to the titers obtained in the control ST cells. For both intra- and extracellular virions, at 24 hpi, the titers of PRV replicating in the ST-LLT [1–3] were approximately 2 times higher, suggesting that the virus could compensate for the suppression of its replication induced by viral miRNAs.

The outcome of this research implies that the overexpression of LLT [1–3] miRNAs affects IE180, EP0, and gE expression, and subsequently, this downregulation has an influence on the replication dynamics. Comparison of the plaque size analysis and the growth kinetics may imply the potential of PRV miRNAs to fine-tune the viral replication, presumably via the control of viral assembly during the early stages of infection.

## 4. Discussion

The biological role of viral miRNAs has been one of the most extensively studied virological issues for nearly two decades. Recent discoveries have indicated that those small regulators can contribute to the repertoire of host-pathogen interactions during viral infection by controlling gene expression at the post-transcriptional level [42,43]. For PRV, several reports have been presented based on the in silico approach, indicating the role of PRV-encoded miRNAs in regulating the virus life cycle [23,25,26]. Two functional studies have explored the importance of a set of PRV miRNAs in pathogenesis and establishment of latency, employing PRV deletion mutants lacking a cluster of nine [44] or 11 miRNAs [28] located within the LLT locus. Experiments validating the function of particular miRNAs in determining the phenotypes of PRV have been limited to a loss-of-function analysis for prv-miR-LLT7-5p [28] or a transient transfection approach with a miRNA mimic for prv-miR-LLT11a [29]. The role of particular fragments of the LLT locus in regulating PRV gene expression and replication has not received thorough attention.

Therefore, we have established a research model based on the stable production of a frontal cluster of three amongst 11 miRNAs mapped to the intron of the LLT [23] in the swine testis (ST) cell line (ST-LLT [1–3]) to unravel the relevance of prv-miR-LLT1-3p (LLT1), prv-miR-LLT2-5p (LLT2), and prv-miR-LLT3-3p (LLT3) during PRV strain NIA-3 lytic infection. We decided to analyze a fragment of the LLT intron-containing precursors of three distinct miRNA genes to ensure the natural genetic context, reflecting natural miRNA biogenesis with respect to the isomiRs formation, and to provide an innate ratio between mature viral miRNAs [45]. The value of our experimental approach lies in the use of an overexpression cell system to highlight the potential of PRV miRNAs in regulating the production of selected proteins and in controlling the viral replication cycle. Most of the recent studies have reported LLT1 as the most abundant viral miRNA throughout the course of PRV infection in the epithelial PK15 cells [25,26] and neuro-2a mice cells [27]. In another study, LLT2 was shown to be the predominant viral miRNA in PRV-infected PK15 cells and swine trigeminal ganglion latent for PRV [44]. The present study analyzed the expression levels of LLT1, LLT2, and LLT3 in PRV-infected ST cells. We observed that LLT1 was most abundantly expressed in productively infected ST cells and that the distribution of those three miRNAs in the course of infection was quite even, except for LLT1, which was predominantly expressed late in infection at 24 hpi (Figure 2D). This result is in line with the proportion of PRV miRNAs expressed in the ST_LLT [1–3] cell line, where LLT1 is also mostly produced (Figure 1C). The expression profiles of miRNAs seemed to fluctuate in the course of infection, which may indicate that individual miRNAs target genes of different kinetic classes. LLT2 and LLT3 are produced from the jointed hairpin precursors, and they may be generated by different mechanisms, affecting their expression levels [23 and Figure 2D]. The divergence in miRNA expression profiles between particular studies may arise from an alternative experimental approach, variability between PRV strains (PRV-Kaplan in [44], PRV-NIA-3, and Begonia in [25], PRV-JS-2012 in [26], PRV-QXX in [27]) and infected cells, and different time points analyzed.

Although PRV encodes miRNAs also within the open reading frames of lytic genes [26], we decided to study miRNAs encoded solely within the LLT, which is antiparallel to and overlaps genes of two important viral transactivators, IE180 and EP0. The present study demonstrated that each of three miRNAs clustered in LLT [1–3] could be detected as early as 3 hpi in ST cells infected with PRV. This result confirms that the expression of PRV-NIA-3 miRNAs located in the LLT transcript occurs during lytic infection. It also indicates their role in the initial phase of the infection process, which is activated by IE180. In silico predictions performed by independent groups have indicated that the IE180 gene is a target for LLT1 [23,24,25] and the EP0 gene is a target for LLT1 [25] and LLT2 [23]. In this study, the obtained data revealed that the overexpression of the frontal cluster of three PRV miRNAs could downregulate the level of IE180 and EP0 mRNAs, which implies that miRNAs act via mRNA degradation. This result is in accordance with the findings of Wang and colleagues, which demonstrate that the PRV-∆miR cluster mutant lacking 11 miRNAs showed higher expression of IE180 at 3 hpi in infected PK15 cells [28]. PK15 cells infected with the PRV-ΔmiR virus could, however, generate higher amounts of EP0 protein (its transcript was not analyzed) at 3 hpi [28]. Differences in the obtained results may arise from another miRNA set being analyzed (the contradictory effect of other miRs within the cluster), different moi of the virus affecting kinetics (moi of 5 versus 1), the virus strain (Chinese PRV-Ea vs. NIA-3), or the cell line model. In the same study, Wang and colleagues have demonstrated that prv-miR-LLT7-5p downregulated the IE180 gene expression, which, in comparison to our results, suggests that various miRNAs from the same primary precursor may cooperate in the regulation of common targets. We cannot exclude that decreased expression of IE180 might also have resulted from the inhibition of its translation. In addition, the determination of whether EP0 is a direct target of PRV miRNAs or whether the effect of LLT [1–3] on its mRNA is indirect and reflects IE180 changes requires further study.

To further determine the role of LLT [1–3] miRNAs in viral gene expression, we asked a question about the effect of LLT [1–3] miRNAs on the expression of an early-late gene coding for a structural protein gE. As mentioned before, gE is an important neurovirulence factor, and in complex with gI, it modulates the cell-to-cell spread mechanism facilitating a direct spread of the infection to neighboring cells and avoiding host immune response [46]. Data collected for transcript and protein levels of gE indicate that the overexpression of LLT [1–3] downregulates the production of this protein at 5 and 7 hpi. However, it remains unclear whether LLT [1–3] miRNAs can directly repress gE production. None of the bioinformatics analyses have indicated gE transcript as a direct target of LLT [1–3] miRNAs. The pattern of gE mRNA inhibition was similar to that of both transactivators, suggesting that LLT [1–3] miRNAs may indirectly affect gE expression. Therefore, the regulatory effects of LLT [1–3] miRNAs on gE during PRV infection may be a consequence of IE180 and EP0 downregulation, which is consistent with the cascade model of gene expression. The observations of gE at the surface of ST_LLT [1–3] cells indicated the contribution of PRV miRNAs in limiting the gE production by 30% at 7 hpi, which in turn implies that PRV miRNAs are able to regulate the level and rate of appearance of viral glycoproteins on the surface of the infected cell.

For the analysis of the impact of LLT [1–3] miRNAs-induced downregulation of viral protein synthesis on the PRV replication kinetics, first, we analyzed the plaque morphology. The plaque size in PRV-infected ST_LLT [1–3] cells was 10% reduced, which could be caused by gE level reduction and cell-to-cell spread impairment. In the previous study, Wang and colleagues analyzed the role of the cluster of 11 PRV miRNAs and single prv-miR-LLT7-5p in plaque formation [28]. They reported that the lack of PRV miRNAs in both cases contributed to the small plaque phenotype. The findings of Wang’s group also indicate that PRV miRNAs affect viral growth kinetics, as the titers of PRV mutant lacking the cluster of 11 miRNAs were lower in a one-step growth curve. In contrast, Liu and colleagues demonstrated that transfection with prv-miR-LLT11a mimic and subsequent PRV infection resulted in inhibition of PRV replication [29]. The observed inconsistency in the estimation of PRV miRNAs effect on viral growth kinetics may result from a different set of miRNAs being analyzed and from various experimental approaches being utilized.

In the present study, we analyzed the replication kinetics of intra- and extracellular PRV in cells overexpressing the LLT [1–3] miRNA cluster. Our data regarding the kinetics of intracellular virion formation indicate a slight delay in virus production in the presence of LLT [1–3] miRNAs observed at 5 and 7 hpi (Figure 5B). At 12 hpi, we could observe the acceleration of viral production, with the following doubled amount of the progeny virions in the ST_LLT [1–3] cells compared to the control cells at 24 hpi. This “catching-up” effect resembles the report by Wang and colleagues, where the IE180 and EP0 levels were altered upon LLT intron deletion only early in infection but became equal as the infection progressed. The suggested explanation for this phenomenon was the accumulation of EP0, induced by PRV miRNAs at the early hours of infection, which was sufficient to drive the course of late infection. Similarly, we suggest that the increase of IE180 and EP0 in LLT [1–3]-expressing ST cells observed between 5 and 7 hpi (Figure 1B,C) could be sufficient to enhance late gene expression and subsequent virion overproduction observed at 24 hpi for both intra- and extracellular viruses. EP0 was denoted in [23] as a potential target of LLT2, which may upregulate EP0 expression via some other mechanisms [47]. These observations support our hypothesis that miRNAs modulate viral infection at the early stages. Interestingly, during the course of the PRV infection of the ST cells, LLT1 significantly accumulated with time, which was not observed for LLT2 and LLT3. We speculate that an increased amount of LLT1 after virus accumulation at 24 hpi may influence host genes, presumably involved in immune response; however, further studies are needed for confirmation. Liu and colleagues reported that prv-miR-LLT11a can inhibit the expression of swine leukocyte antigen (SLA)-1 and transporter associated with antigen processing (TAP) genes at 24 hpi [29]. Because LLT1 is predominantly expressed in the ST_LLT [1–3] cell line throughout the course of PRV infection, we assume that the effects of miRNAs observed in this study may be principally induced by LLT1 action, which stands in line with previous bioinformatics predictions. Further determination of the LLT1 role in PRV infection needs subsequent investigation.

Despite the low statistical significance of changes in replication kinetics observed in PRV-infected ST_LLT [1–3] cells (except for 7 hpi), we speculate that this delay in mature virion production may significantly contribute to virus-host interactions, delaying the cellular immune response. This shift may result from IE180 and EP0 downregulation at the early infection stages, with subsequent alteration in the expression of genes coding for glycoproteins, as we could observe for gE (Figure 4C,D). gE, glycoprotein I, and glycoprotein M were reported to mediate virion maturation [48]. Moreover, gE in complex with gI suppresses type I interferon production in plasmacytoid dendritic cells [49]. Taken together, our findings indicate that PRV miRNAs encoded within LLT [1–3] cluster have the potential to fine-tune the time course of lytic infection and cell-mediated immune response.

## 5. Conclusions

In this study, we generated a cell line that constitutively overexpresses three out of 11 PRV miRNAs mapped to the intron of the LLT locus to study the potential of these miRNAs to regulate the viral gene expression and replication kinetics. We demonstrated that among the LLT [1–3]- clustered miRNAs, prv-miR-LLT1-3p was the most extensively expressed in the ST_LLT [1–3] cell line and in PRV-infected ST cells. The overexpression of miRNAs in the PRV-infected ST_LLT [1–3] cell line resulted in the downregulation of IE180, EP0, and gE genes at the early stages of infection, leading to a slight distortion in transmission and proliferation ability. These findings indicate that LLT [1–3] miRNAs demonstrate the potential to contribute to the modulation of host response via the control of viral glycoproteins appearance at the surface of PRV-infected cells, virion assembly, and viral egress.

## Figures and Tables

**Table 1:** List of primers used in this study. Restriction sites are denoted in bold; n/a: not applicable.

Usage	Primer Name	Sequence (5′→3′); Restriction Enzymes Recognition Sites in Bold	Location (nt) in KU900059
ST_LLT [1–3] construction	PRVmiR1-3BEF	AAT**GGATCC**AGTCCATGACGGTGAGTG	97,486–97,503
	PRVmiR1-3BER	GAC**GAATTC**ACGCTGATACTCTTGGCC	98,670–98,687
miRNA qPCR	qPCR-F-miR-1	TCTCACCCCTGGGTCCGTCGCAA	97,837–97,857
	qPCR-F-miR-2	CTCATCCCGTCAGACCTGCGAA	98,298–98,317
	qPCR-F-miR-3	CGCACACGCCCCTCTCGCGCACAA	98,398–98,419
mRNA qPCR(sequences from [32])	RT2ie180F	CATCGTGCTGGACACCATCGAG	103,891–103,912
	RT2ie180R	ACGTAGACGTGGTAGTCCCCCA	103,844–103,865
	RT2ep0F	GGGTGTGAACTATATCGACACGTC	96,891–96,914
	RT2ep0R	TCAGAGTCAGAGTGTGCCTCG	96,864–96,884
	RT2us8F	CTTCGACGTCTGGTTCCGC	124,454–124,472
	RT2us8R	GGTCACGCCATAGTTGGGC	124,502–124,520
	RT2s28F	GGGCCGAAACGATCTCAACC	n/a
	RT2s28R	GCCGGGCTTCTTACCCATT	n/a

## Data Availability

Not applicable.
